# The Role of Adipocyte Precursors in Development and Obesity

**DOI:** 10.3389/fendo.2020.613606

**Published:** 2021-02-19

**Authors:** Tammy Ying, Rebecca A. Simmons

**Affiliations:** ^1^ The Cell and Molecular Biology Graduate Group, Perelman School of Medicine at the University of Pennsylvania, Philadelphia, PA, United States; ^2^ Institute for Diabetes, Obesity & Metabolism, Perelman School of Medicine, University of Pennsylvania, Philadelphia, PA, United States; ^3^ Department of Pediatrics, Perelman School of Medicine at the University of Pennsylvania, Children’s Hospital of Philadelphia, Philadelphia, PA, United States; ^4^ Center for Research on Reproduction and Women’s Health, Perelman School of Medicine at the University of Pennsylvania, Philadelphia, PA, United States

**Keywords:** development, adipocyte precursor, adult, adipogenesis, adipocyte lineage

## Abstract

Maintenance of adipocyte precursors is critical for regulating metabolism and preventing obesity related disease. These precursors have been immortalized and studied in cellular models as well as—more recently—in animal models. However, little is known about adipocyte precursors from animals of different ages. Most research has focused on adipocyte precursors during obesity. This review goes over the most recent reports of adipocyte precursors during development and in adulthood. Some of these new analyses are due to new techniques such as single cell-RNA sequencing and temporally controlled lineage tracing. With these tools, we have been able to further our understanding of adipocyte precursor lineages and their different regulatory mechanisms. As we learn more about adipocyte precursor plasticity and regulation, we can hope to use this knowledge for future clinical applications.

## Introduction

Once viewed as an inert organ of energy storage, adipose tissue is now appreciated to be a central node for the dynamic regulation of systemic metabolism and energy expenditure. Most research on adipose tissue has focused on mature animals and there is a dearth of information on adipogenesis in developing animals. However, there are a number of critical periods during development that appear to influence the later onset of obesity, in particular the perinatal period. It has long been acknowledged that progenitor cells isolated from neonatal mice differentiated more readily than progenitors from mature animals, perhaps due to an increased percentage of committed pre-adipocytes. However, with the use of temporally restricted lineage tracing *in vivo*, it is becoming apparent that the process of adipogenesis during development is drastically different and unique from adipogenesis in the adult animal.

## White, Brown, and Beige Fat

Adipose tissue includes brown, beige and white fat. Whereas white fat stores energy as lipid, brown fat releases energy as heat. Uncoupling protein 1 (UCP1) is the critical protein required for this thermogenesis in brown adipocytes. Developmentally, cells expressing paired dermomyotomal box protein 7+ (Pax7), essential for the myogenic potential, survival, and proliferation of myogenic progenitors, at E10.5 have been shown to contribute to interscapular brown adipose tissue (BAT) as well as to muscle and dermis (1). The precise developmental origins of individual fat depots during fetal development is still unclear. Experimental evidence suggests that specific anatomical depots arise from distinct lineages. Overall, adipocytes typically are derived from the embryonic mesoderm similar to skeletal muscle, bone and connective tissues. The pool of multipotent progenitors includes engrailed-1 (EN1) positive cells. EN1 is a homeobox gene that regulates midbrain and cerebellum development. Peroxisome proliferator activated receptor gamma (Pparγ) expression is required and sufficient for adipocyte differentiation, making it the key marker identifying committed pre-adipocytes from progenitors and other precursors. In brown fat, Pparγ mRNA expression has been reported at E15.5, Adipoq mRNA expression has been reported as early as E10 and UCP1 protein expression peaks at P10 (2–4). Although BAT has previously been thought of as homogenous, a recent study using single cell RNA sequencing identified two distinct populations characterized in BAT: high and low thermogenesis (4). Although both populations retain morphological characteristics of brown adipocytes (abundant mitochondria, multilocular lipids), the high thermogenesis population expressed high *Ucp1* mRNA, and low thermogenesis expressed low *Ucp1* mRNA.

Previously, it was thought that BAT and white adipose tissue (WAT) were two lineages that diverged developmentally from the same precursor cell type. However, recent evidence suggests that BAT actually develops from a separate cell population than WAT or beige tissue. The BAT precursor population is myogenic factor 5 positive (Myf5), whereas the WAT and beige tissue precursors are platelet-derived growth factor receptor alpha positive (Pdgfrα). *Myf5* encodes a basic-helix-loop-helix transcription factor that initiates the myogenic differentiation program (5). *Pdgfrα* encodes a tyrosine kinase that determines progenitor commitment to beige adipogenesis (6). Therefore, BAT and muscle cells are derived from the same Myf5+ precursor cells and are subsequently interlinked functionally as well (7). WAT and beige precursors derive from the same Pdgfrα+ precursors as fibroblasts, undergoing a completely separate development than BAT. Because white and brown fat come from these separate cell types, *in vivo* lineage tracing must be done using different animal models. This has led to specific work delineating either BAT precursors or WAT precursors with little comparison between the two.

## Fat Development

As mentioned previously, white fat stores energy as lipid. Beige fat can act like white fat and store energy as lipid or like brown fat can release energy as heat. Beige fat becomes thermogenic through transient expression of UCP1, whereas brown fat stably maintains UCP1 (8). White fat is found throughout the entire body but only specific depots are generally dissected and analyzed from the subcutaneous or intra-abdominal/visceral compartments. Wilms tumor 1 (Wt1) is a key regulator of mesenchymal progenitors during kidney and heart development. Interestingly, *Wt1* is expressed in tissues localized near the visceral fat depots, but not WAT or BAT. Knock-in mice expressing tamoxifen-inducable Cre-recombinase driven by *Wt1* promoter activity were crossed with the reporter mTmG mice. In the crossed mouse, Tomato is expressed until Tamoxifen injection, then *Wnt1*-driven Cre cells will express GFP. This effectively marks all *Wnt1* expressing cells at the time of injection. Chau et al. found Wt1+ cells contributed to visceral WAT, but not subcutaneous WAT or BAT. This supports a model whereby individual anatomical depots have distinct origins (9). Interestingly, white adipose depots in mice begin the differentiation process at different developmental timepoints. Inguinal subcutaneous white adipose tissue (iWAT) begins development at E16.5 and gonadal intra-abdominal WAT begins development postnatally in the mouse (10, 11).

Adiponectin (Adipoq) is a secreted hormone that binds multiple tissues and affects obesity related disease. Postnatally, *Adipoq* mRNA expression can only be found in mature adipocytes (10). Surprisingly, the adipocyte precursors in iWAT begin to express *Adipoq* mRNA by E18.5. Using PPARγ induced tracing in mice, adipose precursors have been shown to be specified as early as E10.5 (12). Although BAT and beige fat both similarly express UCP1 and release energy as heat, beige adipocytes only begin differentiating in mice after birth and peak at P21 when kept at room temperature. Under-nutrition during the lactation period has been shown to decrease beige adipogenesis in postnatal day 21 (P21) mice (13). However, this is a transient response and does not affect the amount of inducible beige adipogenesis in adult mice.

## Developmental vs. Adult Adipogenesis

The formation of fat depots in developing animals is a drastically different process than the accumulation of fat in mature animals. The main difference is in the type of fat tissue growth. Hypertrophic growth is due to enlargement of individual adipocytes through lipid expansion. Hyperplastic growth is a result of increased cell numbers (adipogenesis). Hyperplastic growth from adipogenesis can include proliferation of adipocyte precursors and an increased rate of terminal differentiation into mature adipocytes. Neonatal adipose tissue expands through hypertrophy and hyperplasia but adult male iWAT is almost exclusively expanded through hypertrophy (11). This hypertrophic growth is highly correlated to metabolic disease, garnering increased interest in developing a method to induce hyperplastic growth instead. This difference in growth pattern is one of many ways that developmental adipose tissue is distinctive from adult adipose tissue

The process of adipogenesis also has been reported to change with age, although there are only a few studies. The Scherer group found *C/ebpα* is not required for the maturation of precursors in iWAT or perigonadal WAT during postnatal development. However, *C/ebpα* is required for adult adipogenesis (14). The Rodeheffer lab compared adipogenesis in obesity and adipogenesis in neonates, finding that the PI3K-AKT2 pathway is required in obesity-induced expansion of WAT, but not in developmental WAT growth (15).

## Separation of Developmental and Adult Adipocyte Precursors

**Figure 1:**
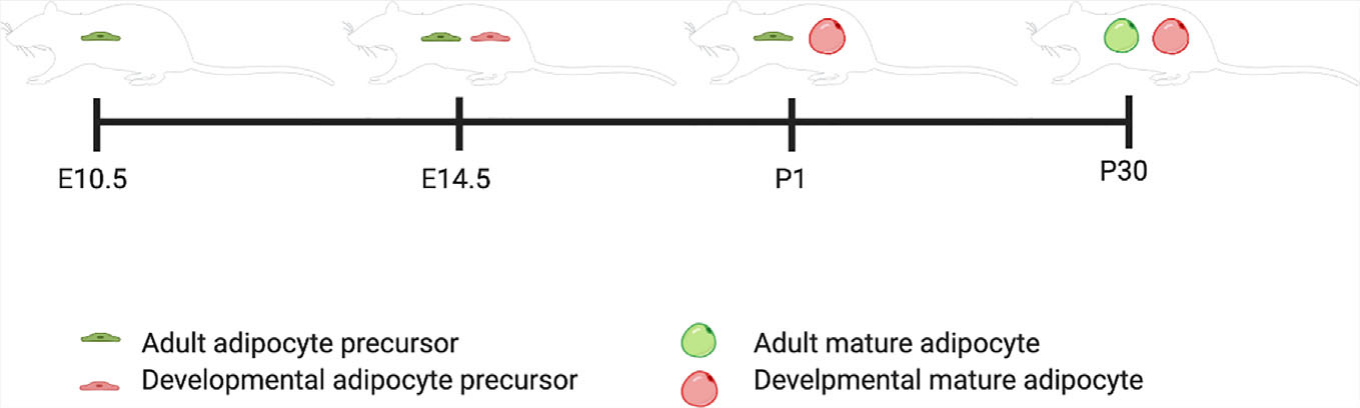
Developmental and adult adipocyte precursor specification timeline. This figure illustrates the timing of adipogenesis for adult versus developmental adipocyte precursors. Created with BioRender.com.

This review will follow the nomenclature the Graff lab has defined: adipocyte precursors that terminally differentiate during the developmental period (E1-P28) are “Developmental” adipocyte precursors and during the adult period are (P28 onward) “Adult” adipocyte precursors. An AdipoTrak system was developed, which is a *PPARγ*-reporter strain that is repressed by Doxycycline. They showed through a time course of AdipoTrak labeling that adult pre-adipocytes are specified to their fate earlier than developmental pre-adipocytes even though they do not go through terminal differentiation until after the developmental pre-adipocytes have all differentiated (**Figure 1**). For adipose tissue that developed within the first 30 days of life, gonadal precursors began expressing PPARγ between P4 and P10 (12). Subcutaneous precursors began expressing PPARγ between E14.5 and P2. The earliest PPARγ+ precursors that were destined to terminally differentiate between P1 and P30 were identified at E14.5 (12). PPARγ+ precursors that developed into adult fat after P30 are PPARγ positive by E10.5 (12).

The major question the Graff lab asked was whether the developing adipocyte precursor population changes over time to become the adult adipose precursor population or if both populations are unique in origin. Using a conditional deletion of *PPAR*γ in the stem compartment that differentiates into adult adipocyte precursors (E0-E10.5), they found no change in phenotype in neonatal mice compared to control mice. However, as the mice aged into adulthood, they had decreased weight and developed glucose intolerance. These results support a model where developing adipose and adult adipose precursor cells are two distinct populations (12).

The adipocyte precursor populations have also been traced *in vivo* using PDGFRα and PDGFRß (fibroblast lineage markers). Through mosaic lineage labeling, it was recently shown that adipocytes formed during the postnatal period are derived from both these lineages, but adipocytes formed during adulthood derive only from the Pdgfrα+ lineage (16). Deletion of *Pdgfr*α and *Pdgfrß* during the perinatal period increases adipogenesis, and deletion of the same genes during adulthood enhances beige adipogenesis. Thus, both signaling pathways decrease adipogenesis during development as well as in adult animals. This suggests that they activate a similar downstream signaling pathway that inhibits adipogenic differentiation. Recently, single cell analyses have also revealed functionally distinct PDGFRß expressing subpopulations in gonadal WAT (17). Finally, data from the Olson lab demonstrate that activation of PDGFRα blocks embryonic white adipose tissue organogenesis in a cell-autonomous manner *via* inhibition of the formation of adipocytes from precursor fibroblasts (16, 18).

Developmental and adult adipose precursors also localize to different anatomical niches which could be due to functional differences in the migratory potential of precursor populations (12). However, angiogenesis and tissue resident macrophages are required for normal development of perigonadal intra-abdominal WAT, suggesting that the micro-environment could also regulate adipocyte precursors (19). For example, a recent study showed that adult adipocyte precursors (PPARγ+) were found at P30 in smooth muscle actin (SMA+) cells in blood vessels. By P60, these precursor cells had differentiated into mature lipid-laden adipocytes. Inducible deletion of PPARγ in SMA+ cells led to impaired glucose tolerance (12). This suggests that SMA+ cells contribute significantly to the adipocyte pool in adult mice and that loss of those adipocytes alter normal metabolic function. Whereas SMA+ adult adipocyte precursors have been localized through lineage tracing to a mural perivascular position, neonatal adipocyte precursors have not. The localization of neonatal adipocyte precursors is still unclear.

## Unique Adipocyte Precursors in Different Niches

In early studies, adipocyte precursors were identified in the stromal vascular fraction (SVF) using *in vitro* culture assays with adipocyte differentiation media selected *against* fibroblasts, immune cells, etc. and selected *for* adipogenic precursors. However, more recently, in order to ensure cell autonomous effects, researchers have been using more stringent protocols to isolate adipocyte precursor populations (20). The question is what defines a particular precursor population? Previous studies used FACs to sort out fibroblasts (CD31+) and immune cells (CD45+) from the SVF and isolate stem cell antigen positive cells (SCA1+). Further purification of CD24+ and CD24- isolated stem cell-like progenitors and committed pre-adipocytes respectively. However, those two populations are still heterogenous to some extent. Single-cell RNA sequencing (scRNA-seq) with unsupervised clustering has become the gold standard for the identification and classification of unique cell populations. Several labs have used this approach in murine adipose tissue, but mainly in mature animals (21).

The Deplancke lab was one of the first to perform scRNA-seq on 208 cells from the SVF of the transgenic mouse DLK1-RFP mouse strain. They used male and female mature mice and identified CD142+ cells in their single cell analyses. Functional studies *in vitro* suggest that they are adipogenesis-regulatory cells (22). These results differ from those of the Seale lab, but this could be due to differences in animals strain and flow sorting strategies. Next, the Granneman lab published scRNA-seq data of 33,663 cells from mature male C57BL6 mice showing that there is different Pparg expression in gonadal WAT adipocyte precursors compared to inguinal WAT adipocyte precursors (23).

**Figure 2:**
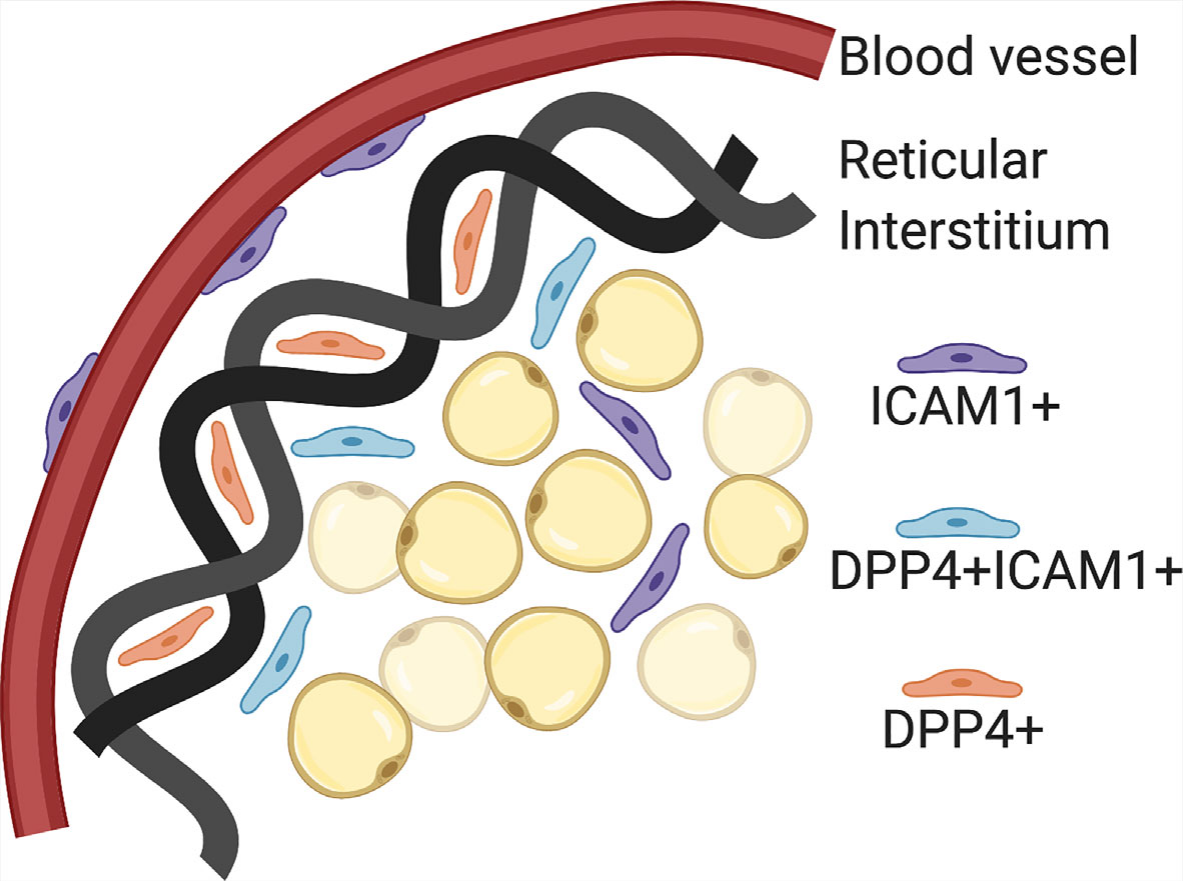
Adipocyte precursor and progenitor niches. Intracellular adhesion molecular 1 (ICAM1+) cells are committed pre-adipocytes, which have been localized to the perivascular space (blood vessels). Dipeptidyl peptidase 4 (DPP4+) cells are pluripotent progenitor cells that have been localized to the reticular interstitium. DPP4+ICAM1+ cells are transitioning from DPP4+ to ICAM1+ and located at the leading edge of the reticular interstitium. Created with BioRender.com.

The Seale lab has published scRNA-seq data on P14 and 8 week old SVF populations in iWAT from C57BL6 mice (24). Using pseudo-time analysis, the reconstruction of cell lineage specification timelines using scRNA-seq data, two pre-adipocyte populations were identified. First, Intracellular adhesion molecular 1 expressing cells (ICAM1+) are canonical committed pre-adipocytes that express PPARγ and easily differentiate with minimal stimulation. Second, CD142+ cells are non-canonical committed pre-adipocytes that readily differentiate but do not express PPARγ. These two originate from the same progenitor, a dipeptidyl peptidase 4 (DPP4+) population that is highly proliferative, multipotent and does not easily differentiate. These DPP4+ progenitors are located in the reticular interstitium, a newly identified niche for adipocyte precursors in mice. The reticular interstitium is a fluid-filled network of collagen and elastin fibers that encases adipose depots and other organs (24). The adipocyte precursors found in this niche express unique lineage markers from those found in the perivascular compartment. If SMA+ progenitors are the cell type that gives rise to adult adipocyte precursors, the next goal in the field is to determine whether DPP4+ progenitors are the population that gives rise to developmental adipocyte precursors. These distinct microenvironments may provide external cues for cellular differentiation and specification *in vivo* (**Figure 2**).

Mural SMA+ progenitors, as mentioned above, have also been described as a precursor to the adult pre-adipocyte. More pseudo-time analyses will have to be done to confirm this possible adipose progenitor cell lineage. As mentioned above, adipocytes are closely related to osteoblasts (bone) and myoblasts (muscle). Therefore, it is not surprising that adipogenesis occurs within muscle and bone as well. A subpopulation of multipotent stem cells from bone marrow has recently been identified and is marked by high expression of Leptin receptor (LepR) (25). *LepR* mRNA expression begins postnatally and LepR+ cells are typically quiescent but become active after injury, irradiation or transplantation (26). They differentiate into osteoblasts as well as adipocytes primarily after 2 months of age, suggesting that this population likely does not contribute to adipogenesis in the developing animal (27).

## Developmental Regulation of Adipogenesis

**Figure 3:**
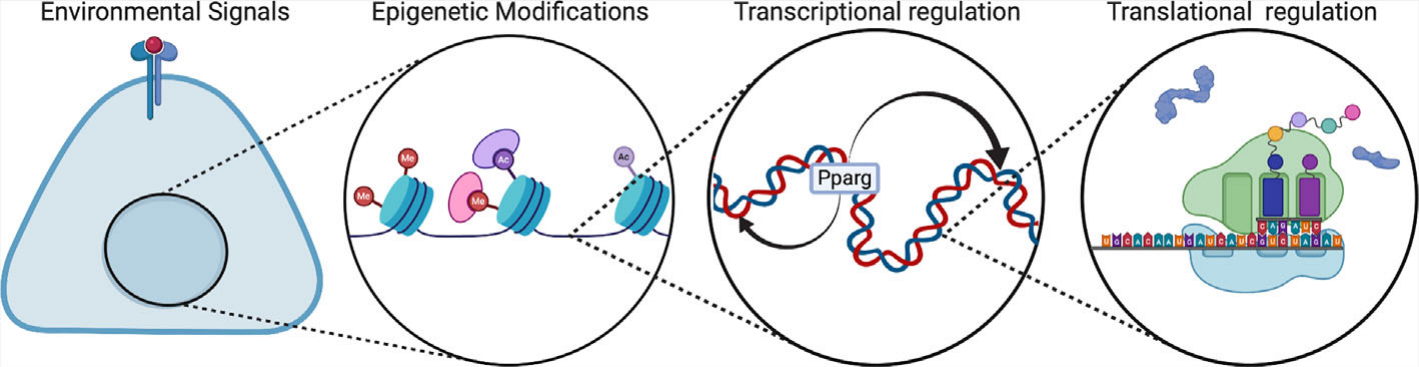
Regulation of multi-level adipogenesis. This figure depicts some of the regulatory mechanisms that have been found to control adipogenesis. These mechanisms can be different in developmental animals compared to adult animals. Created with BioRender.com.

Emerging analyses of regulatory mechanisms during perinatal development show that developmental regulation is distinct from that of mature adipocytes. There are multiple levels of regulation during adipogenesis, including environmental signals, transcription factors, RNA splicing, epigenetic modifications, and microRNAs (**Figure 3**). *In vivo*, the IL-4 receptor signaling pathway has been proposed to be activated in neonates for adipose precursor proliferation and WAT development (28). This pathway is mainly activated by Il-4 released from eosinophils. Although there have been disparate reports, type 2 immunity has been a popular topic of adipogenesis research (28–30). Type 2 immunity is dominant in the newborn and type 2 cytokines such as IL-4, IL-5, IL-33 are expressed at a much higher level compared to adults (31, 32). In addition, type 2 innate lymphoid cells (ILC2) contribute to type 2 immunity and comprised of 3 populations: fetal, neonatal, and adult (33). Most of the ILC2 population in adipose tissue is neonatally derived and undergoes low replenishment with adult ILC2s.

Developmental and adult adipocyte precursors are also regulated differently by transcription factors. In adult obesity models such as high fat diet (HFD), adipocyte precursors have been shown to require activation through the phosphoinositide 3-kinase (P13K)-AKT2 pathway in order to differentiate into mature adipocytes (15). Injection of P13K inhibitors as well as *Pdgfrα-cre; Akt2^flox/flox^* mice show decreased adipogenesis in adulthood during high fat diet feeding compared to controls. At developmental timepoints from P0 to P21, *Akt2^-/-^* mice have similar rates of adipogenesis compared to controls. These data suggest that AKT2 phosphorylation is required for adipogenesis in mature but not developing animals. Another transcription factor, C/EBPα, is also necessary for adipogenesis of white fat in adult animals, but not homeostatic adipogenesis during perinatal development. *In vivo*, C/EBPα targets distinct and separate adipogenesis genes compared to Pparγ, the master regulator of adipogenesis. Defects caused by C/EBPα deletion in adult WAT can be rescued by overexpression of Adipoq. However, the role of Adipoq in adipogenesis is as yet unknown. More recently, the Gupta group has shown that the transcription factor Zfp423 regulates Pparγ in perinatal adipocyte precursors and not mature adipocytes (34). The mechanism of Zfp423 regulation is not well understood, but these studies support the hypothesis that developmental and adult adipocyte precursors are unique populations and that they should be studied as two separate cell types.

UCP1 is temporally regulated by the process of development. Not only is UCP1 protein maximally expressed at P10 in mice, but also splice form regulation of Ucp1 differs between fetal and postnatal development. Splicing does not affect browning but does affect non-shivering thermogenesis (35). Odegaard et al. have shown that this impaired thermoregulation can decrease survival in neonates exposed to cold. There are 2 Ucp1 splice forms, one that becomes protein and one that has an early termination codon and does not get translated. Transitioning from gestational to postnatal life is an environmental challenge that requires activation of thermogenesis to maintain core temperature. Thus, birth has been reported to trigger an increase in the splice form that becomes Ucp1 protein.

Epigenetic regulation has become a focus of adipocyte biology in recent years. It has become clear that adipocyte precursor identity and commitment to differentiation is controlled, at least in part, by epigenetic mechanisms (36). In 3T3-L1 cells, colocalization of C/EBP and PPAR at enhancers open chromatin, which are then enriched with activating histone marks (37). Although epigenetic regulation of adipose lineage genes has primarily been studied in adipose cell lines, some new studies are starting to elucidate epigenetic regulatory mechanisms in primary mammalian cells and *in vivo* (38).

Genome-wide analysis of histone modifications using Chromatin immunoprecipitation combined with sequencing (ChIP-seq) have assessed enrichment of the histone marks responsible for gene silencing (H3K27me3) and activation (H3K4me3) in primary mouse adipocyte precursors in neonates and adults (38). There is also evidence that this regulation differs between adult and developmental adipose precursors. Interestingly, at P14 thermogenic genes in iWAT pre-adipocytes are enriched for both H3K27me3 and H3K4me3 indicating bivalency which is a hallmark of stem-cells (39). Adult (8 weeks) iWAT adipocyte precursors have lower levels of H3K4me3 at these loci, which may partially explain the decrease in adipogenic potential in adult animals compared to neonates (39).

The Ge lab has developed two transgenic mouse lines: one expressing H3.3K36M (a lysine 36 to methionine mutation on histone H3.3) and one expressing H3.3K4M (a lysine 4 to methionine mutation on histone H3.3). The transgenic H3.3K36M mutation blocking H3K36 methylation in early muscle and BAT precursors (Myf5+ cells) showed impaired muscle and BAT development. Inducing the H3.3K36M mutation in mature adipocytes (aP2+ cells) did not affect fat development, but impaired thermogenesis in BAT. They further show that H3.3K36M inhibits adipogenesis by increasing the silencing marker H3K27me3 at adipocyte lineage genes (40). For the transgenic H3.3K4M mutation, the Ge lab show in early muscle and BAT precursors (Myf5+ cells) that it leads to impaired muscle and BAT development. However, inducing the H3.3K4M mutation in mature adipocytes (Adipoq+ cells) induced no change in fat development, maintenance or thermogenesis (41). These data suggest that H3K36 methylation may be required for adipogenesis and thermogenesis, whereas H3K4 methylation is only required for adipogenesis. In the future, crossing these transgenics with conditional reporter mouse strains will isolate out effects of histone modifications on neonatal adipogenesis and adult adipogenesis.

Another regulatory mechanism of adipogenesis that has been studied recently is microRNAs (miRNAs). Clinical studies have shown that circulating miRNAs are dysregulated in obese children (42, 43). However, this area of adipogenesis research is new and there is limited basic research on neonatal animals. One interesting *in vivo* study done in both neonates and adults was on the miR-26 family. The miR-26 family of microRNAs are conserved regulators of adipogenesis and have recently been constitutively deleted using the CRISPR/Cas9 system (44). Analysis of P10 mice show no change in WAT mass, while adult mice at 5 weeks of age show an increase in whole body fat content (44). This suggests that miR-26 acts *in vivo* as a normal suppressor of adult adipogenesis and does not affect developmental fat. Using this study as an example, further studies on miRNAs *in vivo* will need to incorporate neonatal timepoints in order to detect these discrete phenotypes and gain a complete picture of miRNA regulation.

## Developmental Adipocyte Precursors in Human Health and Disease

In humans, adipose depots first appear in the second trimester (45). Interscapular BAT is found in the first few years of life but decreases with age (46). Thermogenic adipocytes are found elsewhere in the body of adults, but there is much debate over whether they are brown or beige adipocytes (47). New results from single cell RNA sequencing seems to suggest these thermogenic adipocytes are beige adipocytes (48). Therefore, there has been a wave of studies on brown adipocytes in recent years with the goal of creating brown adipocytes from human cells which theoretically could be used clinically counter obesity through subcutaneous injections.

Another area of adipocyte biology research that could have widespread impact is the study of individual adipose depots. In humans, obesity caused by the increased mass of intra-abdominal gonadal WAT (gWAT) is most highly linked to metabolic disease (49). This growth is usually through hypertrophy, whereas subcutaneous inguinal WAT (iWAT) in human adults expands predominantly through hyperplasia. Recently there has been single cell analyses of both of these human adipose tissue depots in obese individuals. Interestingly, these data have shown a link between an adipocyte precursor population expressing GPX3 and decreased rates of type 2 diabetes (50).

These different depots are currently mainly studied in mouse models. Human and mouse adipose tissue are similar in that females preferentially accumulate iWAT, while males preferentially accumulate more gWAT (51). However, female adult mice may be better models for the general study of adipogenesis as they expand both iWAT and gWAT, whereas male adult mice mainly have high adipogenesis in gWAT (11, 52). In neonates both males and females undergo hypertrophy and hyperplasia, showing no overt differences.

## Conclusions and Future Directions

Although primary adipocyte precursors isolated from iWAT has been shown to be highly adipogenic *in vitro, in vivo* tracing in mice has shown that that is not strictly true. *In vivo* signaling in response to high fat diet does not increase adipogenesis in iWAT (14). Since it is more physiological, *in vivo* temporally controlled lineage tracing may overtake *in vitro* isolation transcriptomics going forward.

An increasing number of human and mouse studies point to the mural cell compartment of the vasculature as a home for adult adipocyte precursors (12, 53). Unfortunately, this hypothesis has not been supported by pseudo-time analysis of single-cell data. Further studies will need to be done to clarify the linage and localization of these adipocyte precursors.

Distinguishing the developmental and adult pre-adipocyte populations may shed light on factors governing their different physiological properties. Experiments focused on clarifying whether adult and developmental adipocyte precursors have different ontogeny or just diverge with age will be the most important. A main goal will be to manipulate adult adipose tissue towards growth *via* hyperplasia, which is linked to better outcomes than hypertrophy. Finally, there are very limited studies examining the role of an adverse intrauterine milieu on developmental adipogenesis and future studies should be directed on addressing this important area of research.

## Author Contributions

TY wrote the manuscript. RS wrote and edited the manuscript. All authors contributed to the article and approved the submitted version.

## Funding

This work is supported by R01DK114054 (RAS).

## Conflict of Interest

The authors declare that the research was conducted in the absence of any commercial or financial relationships that could be construed as a potential conflict of interest.
